# Identification and characterization of novel PAX8 mutations in Congenital Hypothyroidism(CH) in a Chinese population

**DOI:** 10.18632/oncotarget.14419

**Published:** 2017-01-02

**Authors:** Shiguo Liu, Xueqin Wang, Hui Zou, Yinlin Ge, Fang Wang, Yangang Wang, Shengli Yan, Hongfei Xia, Mingzhao Xing

**Affiliations:** ^1^ Prenatal Diagnosis Center, The Affiliated Hospital of Qingdao University, Qingdao, China; ^2^ Genetic Laboratory, The Affiliated Hospital of Qingdao University, Qingdao, China; ^3^ Department of Biochemistry and Molecular Biology, School of Medicine, Qingdao University, Qingdao, China; ^4^ National Research Institute for Family Planning, Beijing, China; ^5^ Neonatal Screening Center, Jinan Women & Children Medical Healthcare Center, Jinan, China; ^6^ Department of Endocrinology, The Affiliated Hospital of Qingdao University, Qingdao, China; ^7^ Graduate School, Peking Union Medical College, Beijing, China; ^8^ World Health Organization Collaborating Centre for Research in Human Reproduction, Beijing, China; ^9^ Division of Endocrinology, Diabetes & Metabolism, Department of Medicine, The Johns Hopkins University School of Medicine, USA

**Keywords:** congenital hypothyroidism, thyroid dysgenesis, PAX8 mutation, thyroid gene, paired box transcription factor

## Abstract

**Objective:**

Based on mutations in *PAX8* is associated with thyroid dysgenesis. We aim to identify and characterize *PAX8* mutations in a large cohort of congenital hypothyroidism(CH) from thyroid dysgenesis in Chinese population.

**Methods:**

We screened 453 unrelated Chinese patients with CH from thyroid dysgenesis for *PAX8* mutations by sequencing the whole coding regions of *PAX8* on genomic DNA isolated from blood. Cell transfection assays using various vector constructs and induced mutagenesis as well as electrophoretic mobility shift assays were used to investigate the effects of selected mutations on the transcribing and binding activities of PAX8 at the promoters of target genes for *thyroglobulin (TG)* and *thyroperoxidase (TPO)*.

**Results:**

Five *PAX8* mutations were found, yielding a mutation prevalence of 5/453 (1.1%). We selected two mutations in the critical paired domain of PAX8 and generated mutants D94N and G41V. We demonstrated G41V was unable to bind the specific sequence in the promoters of *TG* and *TPO* and activate them. D94N could bind to *TG* and *TPO* promoters and normally activate the *TG* promoter transcription but not the *TPO* promoter transcription. We also demonstrated a dominant negative role of the *PAX8* mutants in impairing the function of the wild-type PAX8.

**Conclusion:**

We for the first time documented the prevalence and characterized the function of *PAX8* mutations in CH in Chinese population. The study specifically demonstrated the role of novel mutations D94N and G41V in impairing the function of PAX8, providing further evidence for genetic *PAX8* defects as a disease mechanism in CH.

## INTRODUCTION

Congenital hypothyroidism (CH), characterized by elevated thyroid-stimulating hormone (TSH) resulting from reduced thyroid function at birth, is the most common congenital endocrine disease, with an incidence of 1 in 2000–4000 newborns [1]. Thanks to the early screening strategies in recent decades, CH is now a highly identifiable and treatable condition at an early patient age. Yet, it is currently still an important cause of mental retardation originated in infants [2]. Thyroid dysgenesis (TD), characterized by development defects of the thyroid gland, accounts for 85% of cases of CH and presents with varying clinical phenotypes. The thyroid gland in thyroid dysgenesis is not visible (agenesis) in 35–40% of cases, small and ectopically located in 30–45% of cases, and eutopically located but severely reduced in size (hypoplasia) in 5% of cases [2]. Thyroid dysgenesis occurs mainly sporadically, with only about 2% being familial [3]. Mutations in the genes for TSH receptor [4], NKX2-1 (thyroid transcription factor 1) [5], thyroid transcription factor 2 [6], and paired box transcription factor 8 (PAX8) [7] have been identified in some patients with various forms of thyroid dysgenesis.

As a major specific regulator of the thyroid gland, the role of *PAX8* in thyroid dysgenesis has drawn particularly considerable attention. PAX8 is a paired domain-containing protein in the mammalian PAX protein family of transcription factors and encoded by a single gene on chromosome 2q12–q14, which consists of 12 exons [8–10]. In addition to the paired domain encoded by exons 3 and 4, PAX8 also contains an octapeptide encoded by exon 5 and a residual paired type homodomain encoded by exon 7 [11, 12]. The 128 amino acids between positions 9 and 137 constitute a sequence-specific DNA-binding domain of the paired domain that is highly conserved within the human PAX protein family [13]. PAX8 is essential both for thyroid development, when the thyroid bud evaginates from the floor of the pharynx, and maintenance of the thyrocyte cell type [14]. This all attributes to *PAX8* being a thyroid-specific transcription factor that regulates thyroid-specific genes, such as thyroperoxidase (TPO), thyroglobulin (TG), and sodium/iodide symporter (NIS), by binding to specific promoter regions via the highly conserved 128-amino acid paired domain [15].

Homozygous *Pax8* knockout resulted in thyroid aplasia in mice [16] and heterozygous loss-of-function *PAX8* mutations were associated with various forms of thyroid dysgenesis in humans [7, 17], genetically implicating the role of *PAX8* defect in thyroid dysgenesis. Most of the inactivating *PAX8* mutations in thyroid dysgenesis have been located in the mutational hotspots of exons 3 and 4 of *PAX8*—corresponding to the DNA-binding paired domain [18–23]. Autosomal dominant transmission occurs for some *PAX8* mutations with variable penetrance and expressivity [7, 17]. Even within the same family, the phenotypes of individuals with heterozygous *PAX8* mutations vary considerably [7, 17, 24].

Even given this knowledge on the role of *PAX8* in thyroid dysgenesis, its genetic molecular pathogenesis has not been uniformly documented and characterized in different ethnic populations, including the large Chinese population. The aim of the present study was thus to screen for *PAX8* mutations in a large cohort of Chinese children with thyroid dysgenesis-caused CH and selectively functionally characterize the novel mutations identified here and in a previous study of ours [25].

## RESULTS

### Identification of novel *PAX8* mutations in TD in the Chinese population

**Figure 1:**
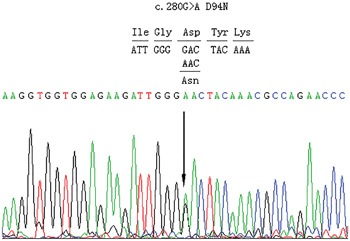
Partial sequences of exon 4 of PAX8 from an affected individual The arrow indicates a heterozygous G>A transition at nucleotide 280 of the coding sequence of PAX8 (c.280 G>A), replacing an invariant aspartate residue by asparaginate at codon 94 in exon 4 (D94N).

**Figure 2:**
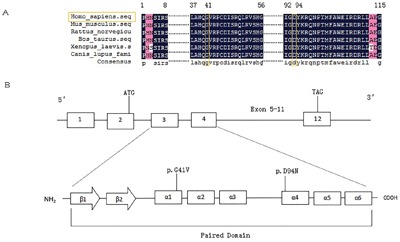
Sequence alignment of PAX8 from different species and schematic representations of the secondary structure elements of PAX8 paired domain **A.** multiple sequence alignment of PAX8 from Homo sapiens, Mus musculus, Rattus norvegicus, Bos taurus, Xenopus laevis, and Canis lupus familiaris. The mutated residues, which are conserved among the different species, are colored in orange at 41 and 94 residues. Less conserved residues are colored in rose. **B.** Schematic representation of the secondary structure elements of PAX8 paired domain and the position of the G41V and D94N mutations in α-helix 3 and 5, respectively. The DNAMAN software was used in these analyses.

We expanded the analysis from our previous small cohort [25] to the current large cohort of 453 unrelated Chinese thyroid dysgenesis patients. We identified three additional novel genetic variants in *PAX8* in this cohort of patients. One was a missense mutation in one patient that predicted an aspartate to asparaginate substitution at codon 94 in exon 4 (c.280G>A/p.D94N) of *PAX8* (Figure 1). Analyses using Polyphen 2 and SIFT revealed that the D94N mutation had a damaging effect on the PAX8 protein, consistent with its being in the pair box domain. We did not find this variant in 100 healthy subjects. The other two variants were PAX8 c.1064C>T/p.A355V (rs145036350) and PAX8 c.-26G>A in two unrelated athyroid patients with thyroid dysgenesis; given the thyroid agenesis in the affected patients, it is possible that the former may impair the PAX8 protein function since it is a non-sense mutation and the latter may affect the *PAX8* gene expression since it is in the promoter area. We previously also found a novel *PAX8* mutation before the expansion of the cohort to the current size, which resulted in Glycine to Valine substitution at codon 41 in exon 3 (c.122G>T/p.G41V) [25]; its location in the paired box domain highly implicated a functional impact on PAX8. Multiple sequence alignment of PAX8 from different species revealed that codon 41 and codon 94 carrying G41V and D94N, respectively, were located in highly conserved regions of PAX8 (Figure 2A), while variant A355V was not located in a highly conserved region. The positions of the G41V and D94N mutations are in α-helix 1 and α-helix 4, respectively (Figure 2B). These mutations segregation with the phenotype within the family was not performed because data was lacking.

### Clinical characteristics of the patients carrying *PAX8* mutations

The patient with the D94N mutation was a male subject. He was born at 38 weeks of gestation from unrelated parents who had no family history of thyroid disease. His birth weight was 2,850 g with no abnormal physical findings. Neonatal screening at 6 days of age revealed a high TSH (267.62 μIU/ml). At 26 days of age, his body weight was 3,500 g and height/length 54 cm, with a persistently elevated serum TSH (280 μIU/ml) and low FT4 (2.06 pmol/L) and free T3 (FT3) (1.78 pmol/L). A 99mTc scan confirmed thyroid agenesis, which prompted the diagnosis of CH. Levothyroxine (L-4) therapy was instituted with the dose adjusted appropriately to maintain serum TSH, FT4, and FT3 within normal ranges. He was found to have elevated TSH upon brief withdrawal of L-4 at 3 years of age, confirming permanent hypothyroidism. At the writing of this paper, the patient was 6 years old with normal intellectual development.

The patient with the G41V *PAX8* mutation was a female subject, born at 38 weeks of gestation from unrelated parents. Her birth weight was 3,600 g and length 51 cm. She was diagnosed with CH by neonatal screening revealing a high TSH (120.5 μIU/ml). On re-evaluation at 14 days of age, she had a persistently elevated TSH (176.1 μIU/ml) and low FT4 (6.5 pmol/L). A 99mTc thyroid scan showed a eutopic normal-sized bilobed thyroid gland (left lobe, 2.5 × 1.4 cm; right lobe, 2.4 × 1.4 cm) with weak fixation. She received appropriate L-T4 therapy and her development was normal; at 2 years of age, she was 80-cm tall and weighed 1,750 g, with normal intellectual development.

### Effects of G41V and D94N mutations on the transcribing activities of PAX8

**Figure 3:**
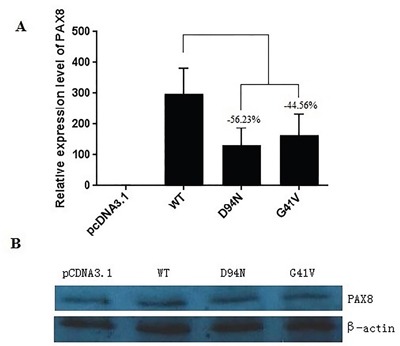
Expression of the wild-type and mutant PAX8 in transfected HeLa cells **A.** After a 48 h of transfection, there was a significant mRNA expression of the wild-type (WT) PAX8 and the mutants. A difference in mRNA level between mutants (PAX8-D94N and PAX8-G41V) and the WT PAX8 by qRT-PCR. **B.** No difference in the expression was found between the WT and the mutant PAX8 proteins (D94N and G41V). β-actin was used to serve as a quantitative protein control.

As G41V and D94N were in the critical functional region of the paired box domain of PAX8, we explored the effects of these mutations on the function of PAX8 protein. We successfully expressed the wild type (WT) and mutant PAX8 in Hela cells. As shown in Figure 3A, quantitative RT-PCR (qRT-PCR) revealed an interesting difference in the mRNA expression between mutant and WT PAX8 mRNA levels; D94N and G41V were 56.23% and 44.56% of that of WT PAX8, respectively. However, Western blotting analysis revealed similar protein expression levels of WT PAX8, G41V and D94N (Figure 3B). These results suggested that even though the mRNA levels of the mutants were somehow lower than WT PAX8, their proteins were efficiently produced to the same levels in this cell system, making the comparable functional studies of the PAX8 mutants in this cell system presented below possible and reliable.

**Figure 4:**
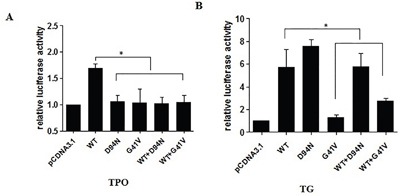
Luciferase activities of mutant PAX8 (D94N and G41V) in HeLa cells cotransfected with PAX8 expression vector (PAX8-WT; PAX8-D94N; PAX8-G41V; PAX8 WT and D94N; PAX WT and G41V) along with TG-Luc or TPO-Luc The ratio between measured firefly and Renilla luciferase activities was expressed relative to the ratio obtained in cells transfected with reporter and empty expression vector (pcDNA3.1) only. **A.** The increase in luciferase activity by WT PAX8 significant compared with G41V, D94N, WT and G41V, WT and D94N group. Failure of the D94N and G41V mutants to activate TPO transcription; Both D94N and G41V had a dominant negative effect on the wild-type PAX8. **B.** The increase in luciferase activity by WT PAX8 significant compared with cells transfected with G41V, WT and G41V. Failure of the mutant PAX8-G41V to activate TG transcription, but normal D94N activation of TG transcription; G41V had a dominant negative effect on the wild-type PAX8. WT, wild type. *P<0.05.

We next examined the effects of G41V and D94N mutants on the transcriptional activities of the target genes *TPO* and *TG*. Specifically, HeLa cells were transfected with expression vectors encoding WT or mutant PAX8, together with reporter gene constructs containing *TPO* or *TG* promoters placed upstream of a coding sequence for the luciferase reporter gene. Compared with WT PAX8, luciferase activities were significantly lower in cells co-transfected with the *TPO* promoter and G41V or D94N (P < 0.05) (Figure 4A). Luciferase activity was not restored by additional cotransfection with WT PAX8. As shown in Figure 4B, when transfected with the *TG* promoter and G41V, cells displayed a much lower luciferase activity than the WT PAX8 (P < 0.001), which could be only partially recovered with additional WT PAX8 cotransfection to a level that is still lower than that achieved with WT PAX8 (P < 0.05). In contract, the ability of D94N to promote the *TG* promoter-driven reporter gene expression was intact. These results thus demonstrated that the ability of G41V and D94N to activate the *TPO* promoter was severely impaired, but only the ability of G41V, but not D94N, to activate the *TG* promoter-driven reporter gene was impaired. These results were also consistent with a dominant negative effect of the two novel PAX8 mutants on WT PAX8.

### DNA binding activities of PAX8 mutants

**Figure 5:**
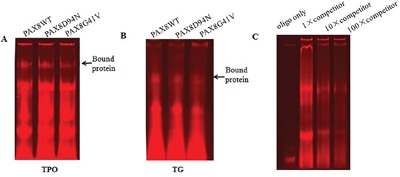
Electrophoresis mobility shift assay with the binding sites in the TPO and TG promoters for mutant PAX8 (D94N and G41V) The arrow indicates the specific binding protein. **A.** The binding to the TPO promoter sequence suggests that PAX8 WT as well as the mutant D94N bound normally to the TPO promoter sequence while G41V lost its binding ability. **B.** In the case of binding to the TG promoter sequence, G41V bound weakly to the PAX8 binding sites of the TG promoter compared to WT and D94N. **C.** The DNA binding is specific for WT PAX8 since a decreased shift can be observed after increasing amounts of unlabeled oligos are added.

We next performed EMSA to investigate the binding capacity of PAX8 mutants to target gene promoters. Oligonucleotides containing the PAX8 binding sequences of the *TPO* and *TG* promoters, respectively, were synthesized and labelled with Alexa Fluor 680. The *in vitro* expressed PAX8 proteins were incubated with the labelled oligonucleotides, separated via PAG, and visualized. We found that mutant D94N, like WT PAX8, could normally bind to the specific DNA sequence in the *TPO* promoter while mutant D41N lost its binding ability (Figure 5A). In the case of the *TG* promoter, G41V could only bind weakly to the PAX8 binding sites compared with WT PAX8, whereas D94N, like WT PAX8, could normally bind the *TG* promoter (Figure 5B).

## DISCUSSION

In this large cohort of thyroid dysgenesis with CH in a Chinese population, we identified several novel *PAX8* mutations and functionally characterized two in functionally critical domain of PAX8—D94N and G41V. Patients with these *PAX8* mutations either had thyroid agenesis or eutopic normal-sized thyroid gland with weak fixation, suggesting that these mutations were the cause of the thyroid dysgenesis in the patients, which were confirmed by our demonstration of the functional defects of the mutants. These cases of patients also show that different *PAX8* mutations may cause different thyroid phenotypes of thyroid dysgenesis, which is well explained by the different effects of D94N and G41V on the transcribing activity and binding ability of PAX8 at the promoters of the target genes *TPO* and *TG*.

Consistent with the present study on Chinese patients, a number of mutations have been reported in the coding regions of *PAX8*, particularly in the paired box domain, which cause loss of function of PAX8 and result in CH with various degree of thyroid dysgenesis in other ethnic populations [7, 24, 27–29]. In this large cohort of 453 Chinese thyroid dysgenesis patients, we identified three novel *PAX8* mutations, including D94N, PAX8 c.1064C>T/p.A355V (rs145036350), and PAX8 c.-26G>A in CH. Together with the other two novel *PAX8* mutations, G41V and R31H, which we had identified in a portion of this cohort of CH [25], the overall prevalence of *PAX8* mutations in the Chinese patients with TD/CH is 1.1% (5/453). This is consistent with the reported low prevalence of *PAX8* mutations in thyroid dysgenesis in other ethnic populations and another Chinese population [24, 26, 30]. For example, in 17 different ethnic cohorts of thyroid dysgenesis patients, the prevalence of *PAX8* mutations was found to be, on average, 1.0%, ranging from 0 to 3.4% [24]. The prevalence of *PAX8* pathogenic variants was 2.38% among patients with CH in another study in Chinese population [26].

The G41V and D94N mutations identified in our study are located in the segment of *PAX8* that encodes the protein homeodomain within the paired domain. Based on the published crystal structure generated using paired domain-DNA complexes [13, 31], the G41V and D94N mutations are located in PAX8 α-helix 1 and 5, respectively, and conserved across various species. As such, it is predictable that the substitution of glycine for valine or aspartate for asparagine, caused by the two mutations, can severely interfere with PAX8 recognition and transcriptional activities of target genes, such as *TG* and *TPO*. This provides a structural basis for our observation that the two mutations indeed severely impaired the transcribing abilities of PAX8 at the target genes *TPO* or *TG*.

Interestingly, D94N showed normal binding to *TPO* and *TG* response elements, yet failed to activate *TPO* promoter-driven reporter gene transcription, and exhibited a dominant negative effect on WT PAX8. However, D94N could activate the *TG* promoter normally. Differences between *TPO* and *TG* promoter structures may explain this disparity in the effect of the PAX8 mutant on the transcriptional activities of the two genes. Binding of G41V to the *TPO* and *TG* promoter binding sites was both impaired, accompanied by expected impaired transactivation of the target gene promoters. Moreover, the ability of WT PAX8 to transactivate the target gene reporter was diminished by mutant G41V co-transfection, exhibiting a dominant negative effect on WT PAX8. It appears that D94N has similar binding and transactivation properties as S48F; the latter is a paired domain mutation of *PAX8* with dominant negative activity and does not transactivate a PAX8-responsive promoter [29]. Another similar example is R133Q, which is located in the highly conserved terminal portion of the PAX8 DNA-binding domain, does not affect the binding ability of PAX8, but exhibits severely impaired activation of the *TG* and *TPO* promoters, although it has no dominant negative effect on WT PAX8 [32]. Our results on G41V are consistent with several previously reported PAX8 mutants, including L16R, F20S, R31C, R31H, Q40P, S54G, L62R, K80_A84dup, and R108X, which all show severely impaired binding to the PAX8 response element and absent transactivational activities on *TPO* and *TG* genes [7, 24, 30]. Our study provides new evidence for a dominant-negative effect of PAX8 mutants, consistent with S48F and S54R [28, 31], but inconsistent with C57Y, D46SfsX24, and R52P which cause CH by haploinsufficiency [17, 30, 33]. The folded PAX8 paired domain functions as a complex nuclear localization signal, which cannot be reduced to a smaller amino acid sequence without loss of nuclear targeting capacity [34]. Hence, inability of PAX8-D94N and PAX8-G41V to activate a PAX8-responsive promoter, despite unimpaired DNA binding of PAX8-D94N *in vitro*, could be due to diminished transport of PAX8-D94N and PAX8-G41V into the nucleus. The possibility should be further confirmed by immunofluorescence in the future study. And The transcription of thyroid-specific genes is dependent on multiple thyroid-specific transcription factors, it will be more helpful if the experiments are repeated in thyroid cell lines.

In conclusion, we have investigated *PAX8* mutations in a large cohort of unrelated Chinese CH patients with thyroid dysgenesis and identified several novel *PAX8* mutations. We extensively functionally characterized two selected mutations G41V and D94N and demonstrated their significant impairing effects on the binding or activating abilities of PAX8 at the promoters of the target genes *TG* and *TPO*, establishing their pathogenic role in the development of CH from thyroid dysgenesis. This is the first report on the prevalence documentation and functional characterization of *PAX8* mutations in a large cohort of patients with CH from thyroid dysgenesis in Chinese population; it is also the largest study on *PAX8* mutations in CH in general. The results also provide new evidence for a dominant negative role of *PAX8* mutants as a disease mechanism in the development of CH from thyroid dysgenesis.

## MATERIALS AND METHODS

### Research subjects

Atotal of 453 thyroid dysgenesis patients (174 boys, 279 girls, sex ratio 1:1.6; age 1.7 ± 0.8 years) diagnosed during neonatal screening for CH in Linyi, Qingdao, and Yantai in Shandong Province, China from 2009 to 2012 were enrolled. TSH (normal range, 0.27–4.2 μIU/ml) and free T4 (FT4) (normal range, 12–22 pmol/L) were determined using standard electrochemiluminescence assay. A diagnosis of CH was established by abnornmally high TSH and low FT4. Thyroid location and size were determined by 99mTc thyroid scan and thyroid ultrasonography, based on which the patients of thyroid dysgenesis were divided into four groups: eutopic and normal-sized thyroid (177 cases, 39%), eutopic hypoplasia (23 cases, 5.3%), agenesis (91 cases, 20%), and ectopic (162 cases, 35.7%). Thyroid dysgenesis patients included in this study were all unrelated individuals with no other endocrine abnormalities, growth failure, or neurological deficits. Blood samples were collected with informed written consent from patients’ parents/legal guardians. The research project was approved by the Ethics Committee of the Affiliated Hospital of Qingdao University.

### DNA sequencing

Genomic DNA from blood samples was isolated using the phenol-chloroform extraction method. Gene fragments covering the coding sequence, the flanking intronic sequence, the 5’UTR, and the 3’UTR of *PAX8* (MIM#167415, GenBank NM_003466.3) were amplified by PCR using conditions and primers described previously [16]. PCR products were purified and sequenced with the BigDye Terminator Cycle Sequencing Kit (Applied Biosystems, Foster City, CA, USA) and run on an automated sequencer, ABI 3730XL (Applied Biosystems) for mutational analysis.

### Constructs and mutagenesis

For functional characterization of *PAX8* mutations, the full coding sequence of *PAX8* was amplified by reverse transcription-PCR from human thyroid mRNA using primers 5′-GGGGTACCATGCCTCACAACTCCATC-3′ and 5′-GCTCTAGACTACAGATGGTCAAAGG-3′. PCR products were cloned into the pCDNA3.1 expression vector (Invitrogen, Carlsbad, CA, USA) using KpnI and XbaI restriction sites introduced into the primers to obtain the wild-type (WT) PAX8-pCDNA3.1( PAX8WT-pCDNA3.1). Mutations were introduced using the Quick Change Mutagenesis kit (Transgene Biotech, Beijing, China), following the manufacturer's protocol, and the following primers: PAX8 D94N-pCDNA3.1 construct, 5′-GTGGAGAAGATTGGGAACTACAAACG-3′ and 5′-TCCCAATCTTCTCCACCACCTTGGGG-3′; and PAX8 G41V-pCDNA3.1 construct, 5′-ACCTGGCCCACCAGGTTGTAAGGCCC-3′ and 5′-ACCTGGTGGGCCAGGTCTACGATGCG-3′.

To create TPO-Luc, a 418-bp fragment containing the human *TPO* promoter was PCR-amplified using primers 5′-GGGGTACCGAGCTGCACCCAACCCAATCCT-3′ and 5′-CCCAAGCTTAGTAATTTTCACGGCTGTAACT-3′. To create TG-Luc, a 305-bp fragment containing the human *TG* promoter was PCR-amplified using primers 5′-GGGGTACCCTTGAGCCTGTTCCCTCCAAAG-3′ and 5′-CCCAAGCTTTTCCTGGCCCTTCCTGGGAGGA-3′. These fragments were cloned via the introduced KpnI and HindIII restriction sites into pGL3-basic (Promega Corp., Madison, WI, USA) to obtain *TPO* prom-pGL3basic and *TG* prom-pGL3basic. All constructs were confirmed by sequencing.

### Cell culture and luciferase assay

HeLa cells were maintained in DMEM supplemented with 1% penicillin-streptomycin and 10% fetal bovine serum under humidified 5% CO2 at 37°C. Cells were plated in 48-well plates 24 hours before transfection. Once the cells reached 90% confluency, transfection using WT or mutant PAX8 was performed using lipidosome 2000 (Invitrogen), following the manufacturer's instructions. For transactivation assays, cells were cotransfected with TG-luc or TPO-luc (250 ng), the internal control vector pRL-CMV (Promega), and PAX8 (200 ng) (WT or mutant). Cells were harvested 48 hours later and lysed in 65 μl lysis solution. To determine the effect of mutant PAX8 on WT PAX8, equal amounts of each mutant vector was cotransfected with WT and reporter vectors. Protein extract (10 μl) was analyzed sequentially for firefly and Renilla luciferase activities using a dual-luciferase reporter assay system (Promega). The total amount of added plasmid was kept constant. The ratio between measured firefly and Renilla luciferase activities was expressed relative to the ratio obtained in cells transfected with reporter and empty expression vector (pcDNA3.1) only. All transfections were performed in triplicate and three independent experiments were similarly performed.

### RT-PCR analysis

Total RNA was isolated from transfected HeLa cells using Trizol agent. Reverse transcriptase reactions were performed in a reaction mixture of 50 ng total RNA, 50 μM oligo(dT)18, and RNase-free water to a final volume of 6 μl. Reaction mixtures were incubated at 70°C for 10 min and then 2 min on ice. This was followed by addition of 10 U RNase inhibitor, 100 U RTase M-MLV, 1x M-MLV buffer, and dNTPs (each at 0.5 mM) (Takara). The reaction mixtures were incubated first at 45°C for 1 hour and then at 70°C for 15 min. Real-time PCR for each cDNA was performed in triplicate in a 10-μl reaction mixture containing 2.5 ng cDNA, 0.2 μl each forward and reverse primer (both 10 mM), and 5 μl SYBR Green PCR master mix (Applied Biosystems). Reactions were incubated in 96-well plates on an Applied Biosystems 7900HT Fast Real-Time PCR system at 94°C for 10 min, followed by 40 cycles of 95°C for 15 s and 65°C for 45 s. The 2-(ΔΔCt) method was used to determine relative quantitative levels of individual cDNAs. GADPH was used for normalization and values were expressed as relative difference compared to corresponding controls. The primers used were: forward, 5′-ACTACAAACGCCAGAAACCCTACCA-3′ and reverse, 5′-TGAATGGTTGCTGCACTTTGGTCC-3′.

### Western blotting analysis

Cell lysates were obtained using protein extraction reagent (Sigma-Aldrich Co. LLC, USA) and protein concentrations determined using the bicinchoninic acid method (Tiangen Biotech Co., Ltd., Beijing, China) with BSA for creating standard curves. Samples containing 30 μg protein were separated on 12% SDS-PAGE gels. Western blotting was performed using rabbit anti-PAX8 primary antibody (1:750; Bioworld Technology, St. Louis Park, USA) and horseradish peroxidase-labeled mouse anti-rabbit secondary antibody.

### DNA binding studies

Electrophoretic mobility shift assay (EMSA) was performed to determine DNA-binding properties of WT and mutant PAX8 using nuclear extracts from transfected HeLa cells. Annealed synthetic oligonucleotide sequences *TPO* (TGATGCCCACTCAAGCTTAGACAG) and TG (CACTGCCCAGTCAAGTGTTCTTGA) were synthesized and 5′-end labeled with Alexa Fluor 680. Binding reactions (10 μl) containing 1 μg protein, 150 ng poly (dI-dC), 500 ng salmon sperm DNA, 5 mM DTT, 0.5% Tween 20, 10 mM Tris, and 50 mM NaCl (pH 7.5) were preincubated at room temperature for 10 min. Double stranded oligonucleotides were then added to a final concentration of 100 nM and incubated at room temperature for 20 min. One μl gel loading buffer (containing 650 mg/ml sucrose, 0.3% Orange G, 10 μM Tris pH 7.5, and 10 μM EDTA pH 8.0) was then added and samples were loaded on 4% native polyacrylamide gels (PAG), supplemented with 50 mM Tris pH 7.5, 200 mM glycine, and 2 mM EDTA. TBE buffer (0.5 X; 0.045mol/L Tris-boric acid and 0.001mol/L EDTA) served as the running buffer. PAGs were scanned and visualized using the Odyssey Model 9120 and Odyssey v2.1 software (LI-COR Biosciences, Bad Homburg, Germany).
